# Kampo Formulae for the Treatment of Neuropathic Pain ∼ Especially the Mechanism of Action of *Yokukansan* ∼

**DOI:** 10.3389/fnmol.2021.705023

**Published:** 2021-12-14

**Authors:** Masataka Sunagawa, Yasunori Takayama, Mami Kato, Midori Tanaka, Seiya Fukuoka, Takayuki Okumo, Mana Tsukada, Kojiro Yamaguchi

**Affiliations:** ^1^Department of Physiology, School of Medicine, Showa University, Tokyo, Japan; ^2^Department of Rehabilitation Medicine, School of Medicine, Showa University, Tokyo, Japan; ^3^Department of Ophthalmology, School of Medicine, Showa University, Tokyo, Japan

**Keywords:** neuropathic pain, analgesic effect, Kampo formula, Kampo medicine, Yokukansan

## Abstract

Kampo medicine has been practiced as traditional medicine (TM) in Japan. Kampo medicine uses Kampo formulae that are composed of multiple crude drugs to make Kampo formulae. In Japan, Kampo formulae are commonly used instead of or combined with Western medicines. If drug therapy that follows the guidelines for neuropathic pain does not work or cannot be taken due to side effects, various Kampo formulae are considered as the next line of treatment. Since Kampo formulae are composed of two or more kinds of natural crude drugs, and their extracts contain many ingredients with pharmacological effects, one Kampo formula usually has multiple effects. Therefore, when selecting a formula, we consider symptoms other than pain. This review outlines the Kampo formulae that are frequently used for pain treatment and their crude drugs and the basic usage of each component. In recent years, Yokukansan (YKS) has become one of the most used Kampo formulae for pain treatment with an increasing body of baseline research available. We outline the known and possible mechanisms by which YKS exerts its pharmacologic benefits as an example of Kampo formulae’s potency and holistic healing properties.

## Introduction

Tricyclic antidepressants, calcium channel α2δ ligands such as gabapentin and pregabalin, and serotonin noradrenaline reuptake inhibitors (SNRIs) are the first choices for treating neuropathic pain according to the Japan Pain Clinic Society’s guidelines for drug therapy (Sumitani et al., 2018). An extract from inflammatory rabbit skin inoculated with vaccinia virus, Neurotropin, and tramadol are second-line treatments. The third-line treatment is potent opioids, such as morphine, fentanyl, and oxycodone. Carbamazepine is the first choice for trigeminal neuralgia, while lamotrigine and baclofen are the second choices. However, the actual efficacy rates of these drugs are low (Finnerup et al., 2015). In many cases, patients have been unable to take them due to side effects. In such cases, Kampo formulae are used as a treatment option in Japan. The Japanese Ministry of Health, Labor, and Welfare has officially approved the clinical use of Kampo formulae as an ethical pharmaceutical. Kampo medicine has been practiced as traditional medicine (TM) in Japan. Kampo medicine uses Kampo formulae that are composed of multiple crude drugs to make Kampo formulae. In recent years, the need for Kampo medicine has increased, and more than 80% of doctors prescribe Kampo formulae clinically (Ito et al., 2012; Moschik et al., 2012). We herein review examples of the clinical uses of Kampo formulae for neuropathic pain and their action mechanisms based on findings in the literature.

## Kampo Formulae for Neuropathic Pain

Kampo formulae are composed of two or more kinds of natural crude drugs, and the decoctions of their mixtures are generally administered. One of the characteristics of Kampo formulae is that they are multi-component formulations, unlike most Western medicines. A single medicine has an analgesic effect along with various other effects such as improving blood flow and coldness while reducing swelling and stress. Therefore, when selecting a Kampo formula, we check for symptoms other than pain.

Table 1 shows examples. For example, patients whose pain is exacerbated by cold stimulation require medicine that relieves pain and simultaneously warms the body. Patients with an impaired blood flow receive medicine that improves the blood flow while reducing pain. Since the factors that make the pain worse can also be improved simultaneously, the therapeutic effect is higher than the administration of analgesics alone. The second feature is that almost no side effects are developed. Kampo medicines have a long history (Kuchta, 2019), and in the process, ineffective and poisonous components have been naturally eliminated. At present, only potent and safe Kampo formulae are cataloged.

**TABLE 1:** Treatment strategies and examples of drug selection in Kampo medicine.

Characteristic symptoms other than pain	Treatment strategies	Representative example of Kampo formulae	References
Cold	Warm	Goshajinkigan (GJG)	Takayama et al., 2018; Matsubara et al., 2021
Heat/inflammation	Cool/anti-inflammatory	Eppikajutsuto (EJT)	Kogure et al., 2013; Shinkai et al., 2017
Microangiopathy	Improving blood flow	Keishibukuryogan (KBG)	Endo et al., 2008; Fujita et al., 2010
Dropsy/abnormal water metabolism	Improving water metabolism	Goreisan (GRS)	Yano et al., 2017; Murakami et al., 2021
Stress/anxiety	Antistress/antianxiety	Yokukansan (YKS)	Katahira et al., 2017; Wada et al., 2017
Decreased physical strength/immune deficiency	Improving physical fitness/improving immunity	Juzentaihoto (JTT)	Ishikawa et al., 2017; Takaku et al., 2020

Table 2 lists the Kampo formulae frequently used to treat chronic pain, their crude drugs, and each crude drug’s main actions and components, classified according to their effects based on Kampo medicine. (TM) has been added to certain terms to indicate that their meaning in this content is in relation to traditional Kampo medicine. Crude drugs with mainly analgesic and anti-inflammatory effects are assigned to Class 1, those with anti-stress effects are assigned to Class 2, and those that improve blood (TM) disturbances are assigned to Class 3. Blood (TM) is a red fluid that supports the nutrition and metabolism of the body, and its disturbance patterns include static blood (TM) and blood (TM) deficiency (The Editing Committee for the Dictionary of Kampo Medicine, the Japan Society for Oriental Medicine, 2020). In addition, crude drugs that enhance the digestive function and improve physical strength are assigned to Class 4, whereas those that improve water metabolism, suppress swelling, and confer a diuretic effect are assigned to Class 5. Class 4 includes crude drugs that have a qi (TM)-tonifying effect. Qi is a fundamental energy required for life activities. Class 5 includes drugs that have a fluid (TM)-regulating effect. Fluid is a colorless fluid that supports nutrition and metabolism including interstitial fluid and lymph. Weights (g) indicate the amount of each crude drug used to produce each Kampo formula, and the drugs marked with (*) are those that play the most central role in each Kampo formula (Takayama, 2019). Aside from Goshajinkigan (GJG) and Yokukansan (YKS), the contents of crude drugs differ depending on the manufacturers, so some patterns were shown. These doses of crude drugs are mixed, and the decoction is administered.

**TABLE 2:** Kampo formulae for chronic pain and crude constituent drugs.

Classification	Crude drugs				Kampo formulae															References
	Latin name	English name	Main effects	Major component	GJG	EJT		KBG		GRS			YKS	JTT		KJT	SKT			
						(1)	(2)	(1)	(2)	(1)	(2)	(3)		(1)	(2)		(1)	(2)	(3)	
1	Aconiti radix processa	Processed Aconiti root	Analgesia, cardiotonic, warm	Aconitine	1.0											0.5 or 1.0				Yu et al., 2012; Deng et al., 2021; Qiu et al., 2021.
	Glycyrrhizae radix	Glycyrrhiza	Analgesia, antiinflammation, antitussive	Glycyrrhizin		2.0	2.0						1.5	1.0	1.5	2.0	1.5	1.5	2.0	Kamei et al., 2005; Wang et al., 2015
	Paeoniae radix	Peony root	Analgesia, antiinflammation, improving static blood (TM), sedation	Paeoniflorin				3.0	4.0					3.0	3.0	4.0	2.0	2.5	2.0	Li et al., 2014; Yin et al., 2016; Xin et al., 2019.
	Cinnamomi cortex	Cinnamon bark	Analgesia, antiinflammation, perspiration, warm	Cinnamaldehyde	1.0			3.0	4.0	3.0	2.0	1.5		3.0	3.0	4.0*	2.5	2.5	2.0	Iwasaki et al., 2008; Churihar et al., 2016; Lee et al., 2018.
	Gypsum fibrosum	Gypsum	Antiinflammation, sedation	Calcium sulfate		8.0	8.0													Liu et al., 2021.
	Ephedrae Herba	Ephedrae Herb	Antiinflammation, perspiration, antitussive	Ephedrine		6.0*	6.0*													Miyagoshi et al., 1986; Wu et al., 2014; Cheng et al., 2017.
	Scutellariae radix	Scutellaria root	Antiinflammation	Baicalin													2.0	2.0	2.0	Shimizu et al., 2018.
2	Pinelliae tuber	Pinellia tuber	Antistress, sedation, antitussive	Homogentisic acid													4.0	4.0	4.0	Goto et al., 2013; Lin et al., 2019.
	Bupleuri radix	Bupleurum root	Antistress, antiinflammation, analgesia	Saikosaponin									2.0				5.0*	5.0*	5.0*	Shin et al., 2019; Guo et al., 2020; Xu et al., 2021.
	Uncariae uncis cum ramulus	Uncaria hook	Antistress, vasodilation, analgesia	Rhynchophylline									3.0*							Pengsuparp et al., 2001; Loh et al., 2017; Qiao et al., 2021.
3	Persicae semen	Peach kernel	Improving static blood (TM), antiinflammation	Amygdalin				3.0*	4.0*											Hao et al., 2019; He et al., 2020.
	Moutan cortex	Moutan bark	Improving static blood (TM)	Paeonol	3.0			3.0*	4.0*											Hirai et al., 1983.
	Rehmanniae radix	Rehmannia root	Tonifying blood (TM), analeptic	Catalpol	5.0*									3.5	3.0					Leong et al., 2018; Wu et al., 2019.
	Angelicae acutilobae radix	Japanese angelica root	Tonifying blood (TM), analeptic	Ligustilide									3.0	3.5	3.0					Shimizu et al., 1991; Hatano et al., 2004.
	Achyranthis radix	Achyranthes root	Improving static blood (TM), improving of fluid (TM), analgesia	Ecdysterone	3.0															Luo et al., 2009; Jung et al., 2015; He et al., 2017.
	Cnidii rhizoma	Cnidium rhizome	Tonifying blood (TM), analgesia, analeptic	Cnidilide									3.0	3.0	3.0					Choi et al., 2016; Lee et al., 2016; Ningsih et al., 2020.
4	Ginseng radix	Ginseng	Tonifying qi (TM), analeptic, stomachic	Ginsenoside										2.5*	3.0*		2.0	2.0	2.0	Zhang et al., 2019; Fan et al., 2021; Qu et al., 2021.
	Corni fructus	Cornus fruit	Tonifying qi (TM), analeptic	Loganin	3.0															Dong et al., 2018.
	Dioscoreae rhizoma	Dioscorea rhizome	Tonifying qi (TM), analeptic, antitussive	Diosgenin	3.0															Kim et al., 2012.
	Zizyphi fructus	Jujube	Tonifying qi (TM), analeptic, antistress	Zizyphus saponin		3.0	3.0									4.0	2.0	2.0	2.0	Peng et al., 2000; Irshad et al., 2020; Wang et al., 2020.
	Zingiberis rhizoma	Ginger	Stomachic, warm	Gingerol		1.0	0.8									1.0	0.5 or 1.0	1.0	2.0	Yoshikawa et al., 1994; Chang et al., 1995; Chrubasik et al., 2005.
	Astragali radix	Astragalus root	Tonifying qi (TM), analeptic, cardiotonic	Formononetin										2.5	3.0					Zhang et al., 2011; Wei et al., 2021.
5	Atractylodis rhizoma	Atractylodes rhizome	Improving static blood (TM), anti-edema, stomachic	Atractylon						4.5^#^	3.0		4.0^#^	3.5	3.0^#^					Hwang et al., 1996; Shi et al., 2019; Zhang et al., 2021.
	Atractylodis lanceae rhizoma	Atractylodes lancea rhizome	Improving of fluid (TM), anti-edema, stomachic, perspiration	Atractylodin		4.0	4.0			4.5^#^		3.0	4.0^#^		3.0^#^	4.0				Yamahara et al., 1990; Koonrungsesomboon et al., 2014; Yu et al., 2017.
	Alismatis tuber	Alisma tuber	Improving of fluid (TM), anti-edema	Alisol	3.0					6.0	5.0	4.0								Makino et al., 2002; Han et al., 2013.
	Poria	Poria sclerotium	Improving of fluid (TM), anti-edema, stomachic, antistress	Eburicoic acid	3.0			3.0	4.0	4.5*	3.0*	3.0*	4.0	3.5	3.0					Nukaya et al., 1996; Lee et al., 2012; Lu et al., 2021.
	Polyporus	Polyporus sclerotium	Improving of fluid (TM), anti-edema, antiinflammation	Ergosterol						4.5	3.0	3.0								Sun and Yasukawa, 2008; Zhang et al., 2010.
	Plantaginis semen	Plantago seed	Improving of fluid (TM), anti-edema, antiinflammation, antitussive	Aucubin	3.0															Park and Chang, 2004; Tzeng et al., 2016; Li et al., 2020.

All crude drugs are listed in the 17th edition of the Japanese Pharmacopeia (Pharmaceutical and Medical Device Regulatory Science Society of Japan, 2017). Class 1, crude drugs with analgesic and anti-inflammatory effects; Class 2, drugs with anti-stress effects; Class 3, drugs with blood flow-improving effects; Class 4, drugs that enhance the digestive function and improve physical strength; and Class 5, drugs that improve water metabolism, suppress swelling, and confer a diuretic effect. Traditional medicine (TM) is added to the terms used in the content of traditional Kampo medicine. Weights (g) indicate the amount of each crude drug to produce each Kampo formula, and the crude drugs marked with (*) and bold are the most active components of each medicine (Takayama, 2019). Except for GJG and YKS, the contents of crude drugs differ depending on the manufacturers, so some patterns were shown. One of the crude drugs marked with (^#^) (Atractylodis rhizoma or Atractylodis lanceae rhizoa) is used. GJG, Goshajinkigan; EJT, Eppikajutsuto; KBG, Keishibukuryogan; GRS, Goreisan; YKS, Yokukansan; JTT, Juzentaihoto; KJT, Keishikajutsubuto; SKT, Saikokeishito.

### Goshajinkigan (Chinese Name: Niu Che Sen Qi Wan)

Goshajinkigan was first described in *Ji Sheng Fang* published in 1253 in China (Yan and Liu, 2012). It is a well-balanced combination of Class 3–5 crude drugs plus Aconiti radix processa and Cinnamomi cortex, which have strong analgesic and warming effects. This combination is suitable for patients who have a decreased physical function, are extremely tired, and complain of coldness, especially in the lower limbs, a dry mouth, and dysuria. GJG is often prescribed for inferior limb pain and lower back pain (Hamaguchi et al., 2017). Recent reports have suggested that GJG may prevent chemotherapy-induced peripheral neuropathy (Nishioka et al., 2011; Cascella and Muzio, 2017).

### Eppikajutsuto (Yue Bi Jia Zhu Tang)

Eppikajutsuto (EJT) was first described in *Jin Gui Yao Lue* published around 200 AD in China (Zhang, 2020). EJT mainly includes Class 1 crude drugs, such as Ephedrae Herba, Gypsum fibrosum, and Glycyrrhizae radix, which have anti-inflammatory activities, and Atractylodis lanceae rhizoma, which improves an uneven distribution of water, such as in case of edema. EJT is useful for relieving edema and knee effusion caused by allergies and inflammation, especially rheumatoid arthritis (Kogure et al., 2013). Since EJT has a very strong anti-inflammatory effect, it is an alternative to non-steroidal anti-inflammatory drugs in patients with gastrointestinal disorders.

### Keishibukuryogan (Gui Zhi Fu Ling Wan)

Keishibukuryogan (KBG), which was first described in *Jin Gui Yao Lue* (Zhang, 2020), is a Kampo formula that improves various symptoms caused by a decreased blood flow and stagnation (Nozaki et al., 2007; Tomita et al., 2017). It is composed of Persicae semen and Moutan cortex, which belong to Class 3, and Paeoniae radix, which improves the blood flow. In addition, the analgesic and anti-inflammatory effects of Paeoniae radix and Cinnamomi cortex, which belong to Class 1, are also present. The administration of KBG is reported to warm diseased limbs and improve post-stroke cold sensations and numbness in the affected body parts by increasing the peripheral blood flow (Fujita et al., 2010).

### Goreisan (Wu Ling San)

Goreisan (GRS) was first described in *Jin Gui Yao Lue* (Zhang, 2020) and *Shang Hang Lun* published around 200 AD in China (Zhang, 1999). GRS consists of Atractylodis lanceae rhizoma (or Atractylodis rhizoma), Alismatis tuber, Poria, and Polyporus (Class 5), which relieve water retention in such conditions as edema, oliguria, and diarrhea and Cinnamomi cortex, which has analgesic, anti-inflammatory, and warming effects. GRS is administered to patients with exacerbated pain due to swelling. Changes in barometric pressure that accompany weather changes can exacerbate pain, and GRS is effective in such cases (Kurihara et al., 2018).

### Yokukansan (Yi Gan San)

YKS was first described in a Chinese medical book *Bao Ying Cuo Yao* published in 1556 (Kai and Ji, 2016). One characteristic of YKS is that it is mainly composed of Bupleuri radix and Uncariae uncis cum ramulus (Class 2), which have anti-stress effects. In addition, it contains crude drugs from Classes 3 to 5. It is useful for patients with a weak constitution, especially those with frustration and anger due to increased sensitivity to stress. Originally, YKS was administered to patients with symptoms of emotional irritability, neurosis, and insomnia and to infants suffering from night crying and convulsions (de Caires and Steenkamp, 2010). The crude drug components of YKS, including Glycyrrhizae radix, Bupleuri radix, Uncariae uncis cum ramulus, and Cnidii rhizome, have analgesic effects. Thus, YKS is also used to treat various pain disorders, including fibromyalgia, post-herpetic neuralgia, phantom-limb pain, headache, and trigeminal neuralgia (Nakamura et al., 2009; Yamaguchi, 2015; Sugasawa, 2016; Akiyama and Hasegawa, 2019). Many studies have been published concerning the mechanism of analgesic action of YKS. Chronic pain causes stress, and stress further promotes and exacerbates pain (Hannibal and Bishop, 2014). YKS is effective in such cases.

### Juzentaihoto (Shi Quan Da Bu Tang)

Juzentaihoto (JTT) was first described in *Taiping Huimin Heji Ju Fang* published (1151) in China (Ping et al., 2017). Long-lasting pain deprives patients of physical strength and reduces their willingness to fight illness. Chronic pain may alter immune response, which can affect recovery from chronic pain (Herzberg et al., 1994; Sunagawa et al., 2000; Bethea and Fischer, 2021). The main components of JTT, Ginseng radix, and Astragali radix (Class 4) improve fatigue, malaise, loss of appetite, and weakened immunity. JTT should improve physical strength to fight illness (Yamakawa et al., 2016). In addition, JTT contains crude drugs from Classes 3 to 5. Glycyrrhizae radix, Paeoniae radix, and Cinnamomi cortex, which have analgesic effects, contribute to a well-balanced formula. JTT is frequently used for cancer patients because it enhances immune function (Saiki et al., 2017; Ogawa-Ochiai et al., 2021).

### Keishikajutsubuto (Gui Zhi Jia Zhu Fu Tang)

Keishikajutsubuto (KJT) was produced by Japanese doctor Todo Yoshimasu (1702–1773) and described in *Hoki* (Yoshimasu, 1181). KJT is mainly composed of Class 1 drugs with anti-inflammatory and analgesic effects. In addition to its strong analgesic effect, Aconiti radix processa, Atractylodis lanceae rhizoma (Class 5), Cinnamomi cortex (Class 1), and Zingiberis rhizoma (Class 4) variously offer warming and diuretic effects. KJT is effective for joint pain and neuralgia associated with coldness and swelling (Nakanishi et al., 2012). Although the crude constituent drugs are similar to EJT, EJT contains Gypsum fibrosum and Ephedrae Herba, which have strong anti-inflammatory effects, and treats cases without coldness.

## Kampo Formulae for Trigeminal and Glossopharyngeal Neuralgias

In Western medicine, treatment strategies differ between trigeminal neuralgia and neuralgia in other parts of the body. Similarly, the drugs used in Kampo medicine are slightly different. Trigeminal neuralgia is divided into idiopathic trigeminal neuralgia caused by the compression of blood vessels around the nerve and symptomatic trigeminal neuralgia caused by organic diseases, such as tumors, other than vascular compression. Drug treatment is less invasive than surgery and is often the first treatment choice, including to achieve pain relief before surgery. In addition, drug administration is performed when surgical therapy, radiation therapy, and nerve block cannot be performed, or when symptoms recur. As mentioned above, the first-line drug is carbamazepine, an antiepileptic drug, but its number needed to harm is 3.4 (Cruccu et al., 2008). Its side effects, including gastrointestinal symptoms, light-headedness, drowsiness, drug eruption, and myelosuppression, also cause dose reduction or discontinuation of administration. In such cases, the carbamazepine dosage may be reduced by concomitant use of Kampo formulae. Kampo formula treatment is a useful countermeasure against side effects caused by long-term carbamazepine use. Frequent treatments for trigeminal neuralgia include GRS (Kido et al., 2017), Saikokeshito (SKT) and KJT.

Glossopharyngeal neuralgia is paroxysmal pain induced by coughing, swallowing, mastication, conversation, and yawning. It occurs mainly in the back of the ear, behind the tongue, tonsils, and pharynx and just below the lower jaw angle. The incidence is reportedly 0.2/100,000, making it a very rare disease. GRS is a common glossopharyngeal neuralgia treatment that it seems to confer anti-inflammatory effects and helps reduce edema around the nerve.

The reason GRS works for trigeminal and glossopharyngeal neuralgias is unclear. However, according to oriental medical theory, neuralgia is caused by the swelling of nerves. Therefore, GRS, which has a diuretic effect, is effective against these neuralgias. KJT would be better for cases with strong symptoms of coldness.

### Saikokeshito (Chai Hu Gui Zhi Tang)

Saikokeshito was first described in *Jin Gui Yao Lue* (Zhang, 2020) and *Shang Hang Lun* (Zhang, 1999). SKT is usually given to patients with cold accompanied by gastrointestinal symptoms. Still, most crude drugs such as Glycyrrhizae radix, Paeoniae radix, Cinnamomi cortex, Scutellariae radix, and Bupleuri radix have analgesic and anti-inflammatory effects. SKT has been reported to exert analgesic activity in a rat trigeminal neuralgia model (Sunagawa et al., 2001). Some reports indicate the efficacy of SKT for epilepsy (Aimi et al., 1976). Therefore, SKT may have an anticonvulsant effect and may be effective for trigeminal neuralgia.

## Action Mechanisms of Yokukansan for Neuropathic Pain

Kampo medicines have a long history, and although their effectiveness has been empirically recognized, their mechanisms of action have not been completely clarified. However, in recent years, basic research on the Kampo formula has been actively conducted, and evidence based Kampo medicine treatments are also being carried out. For physicians who are trained under Western medicine, evidence-based drug selection is more familiar and easier to understand than narrative-based ones. In this section we will consider the mechanism through which Kampo formulae exert their analgesic effects, using YKS as an example.

YKS has been found clinically effective for diseases with chronic pain, including post-herpetic neuralgia, central post-stroke pain, post-traumatic spinal cord injury pain, thalamic syndrome, complex regional pain syndrome (CRPS; Nakamura et al., 2009), trigeminal neuralgia (Yamaguchi, 2015; Takinami et al., 2017), phantom pain (Sugasawa, 2016), migraine (Akiyama and Hasegawa, 2019), and headache (Kimura et al., 2008). Mitsuhata et al. (2010) administered YKS to 121 patients with chronic pain who did not respond to conventional drug therapy or nerve block treatment and found it effective in 73 patients (60%). They also found YKS to be effective in 25 of 47 chronic lumbar and inferior limb pain cases (53%), 3 of 6 cervical or lumbar post-surgery syndrome cases (50%), 13 of 20 post-herpetic neuralgia cases (65%), 6 of 8 herpes zoster neuralgia cases (75%), 7 of 15 cervical spondylosis/cervical spondylotic radiculopathy cases (47%), 2 of 4 perineal pain cases (50%), and 6 of 6 CRPS cases (100%). Considering that all of these entities are intractable painful diseases, the efficacy rate seems to be relatively high.

The analgesic effect of YKS has been proven in some animal models, including a chronic constriction injury (CCI) model (Suzuki et al., 2012; Suga et al., 2015), partial sciatic nerve ligation (PSL) model (Ebisawa et al., 2015), bone metastasis model (Nakao et al., 2019), and adjuvant arthritis model (Honda et al., 2013). Several factors are involved in the complex development and promotion of neuropathic pain. Increased reactivity of the dorsal horn of the spinal cord, i.e., central sensitization, is considered one cause of hyperalgesia and allodynia. Central sensitization includes the following: (1) enhancement of excitatory synaptic transmission, (2) attenuation of inhibitory synaptic transmission, (3) activation of glial cells, and (4) dysfunction of the descending pain modulatory system.

### Enhancement of Excitatory Synaptic Transmission and Yokukansan

From the terminal of the primary afferent nerve C-fiber, neurotransmitters like glutamate and substance P act on each receptor in the dorsal horn of the spinal cord. Continuous or repetitive stimulation from the primary nerve promotes excitatory synaptic transmission by activating and phosphorylating the glutamate receptor, *N*-methyl-D-aspartate (NMDA) receptor.

YKS was observed to attenuate excessive glutamate release from presynaptic sites (Takeda et al., 2008). The removal of glutamate in the synaptic cleft is mainly carried out by the two glutamate transporters in astrocytes: glutamate transporter 1 (GLT- 1) and glutamate/aspartate transporter. YKS has been reported to promote the GLT-1-mediated uptake of glutamate using cultured astrocytes (Ueki et al., 2018). This action appears to be due to Glycyrrhizin and its metabolite, 18β-glycyrrhetinic acid, as well as a compound found in Glycyrrhizae radix (Kawakami et al., 2010). Furthermore, YKS has an antagonistic effect on the NMDA receptor. Isoliquiritigenin, a component of Glycyrrhizae radix, acts as the antagonist (Kawakami et al., 2011). Thus, YKS may suppress excessive neurotransmission mediated by glutamate. Suzuki et al. (2012) reported that YKS inhibited mechanical and cold allodynia in the rat CCI model and reduced the cerebrospinal fluid dialyzate level of glutamate increased by stimulation with a brush or acetone.

### Attenuation of Inhibitory Synaptic Transmission and Yokukansan

The hypofunctions of GABAergic neurons, which are inhibitory interneurons in the spinal dorsal horn, occurred in rodents with chronic pain (Fu et al., 2017). YKS has been reported to reverse the reduction in pentobarbital-induced sleep durations in socially isolated mice (Egashira et al., 2011). It also exhibited anxiolytic effects (Kamei et al., 2009), which are thought to be mediated by GABA_A_ receptors. Liao et al. (1995) reported that the water extract of Angelicae acutilobae radix binds to GABA_A_ receptors *in vitro*. These findings suggest that YKS can be expected to exert an inhibitory effect on synaptic transmissions *via* the GABAergic neuron.

### Activation of Glial Cells and Yokukansan

In animal models of schizophrenia (Furuya et al., 2013), multiple sclerosis (Nomura et al., 2017), and behavioral and psychological symptoms of dementia (Ikarashi et al., 2009), YKS suppresses glial cell (microglia and astrocytes) activity. The activation of these glial cells is associated with the development and persistence of neuropathic pain (Tsuda, 2018), so glial cells and their associated molecules became the targets of YKS treatment. Suga et al. (2015) reported that the administration of YKS inhibited the expression of activated astrocytes and astrogliosis in the CCI rat model. Ebisawa et al. (2015) reported that YKS inhibited the increased expression of interleukin-6 mRNA in the dorsal horn of the spinal cord in the PSL mouse model, and the expression was confirmed in astrocytes and/or microglia, not in neurons. Furthermore, only the administration of Atratylodis Lanceae rhizoma exhibited the same effect. These studies suggest that YKS is effective against neuropathic pain, as evidenced by the regulation of microglial and astrocytic functions, which indicate the formula’s potential mechanisms.

### Dysfunction of the Descending Pain Modulatory System and Yokukansan

Descending neurons from the rostral ventromedial medulla mainly secrete serotonin. In contrast, neurons from the locus ceruleus secrete noradrenaline. Serotonin acts on 5-HT_1A_ and 5-HT_1B_ receptors, which are suppressive serotonin receptors in spinal dorsal horn neurons. Noradrenaline acts on α2 receptors, which suppress synaptic transmission. SNRI treats chronic pain with the expectation that this effect will be enhanced. Dysfunction of the descending pain modulatory system reportedly involves the development of chronic pain (Ossipov et al., 2014). YKS acts as an agonist of the 5-HT_1A_ receptor; geissoschizine methyl ether, an alkaloid synthesized by the YKS component Uncariae uncis cum ramulus, is believed to play this role (Nishi et al., 2012; Yamaguchi et al., 2012). However, whether or not YKS improves the dysfunction of the descending pain modulatory system is unclear, so further studies are needed.

### Other Actions of Yokukansan

The pre-administration of YKS attenuated the development of morphine antinociceptive tolerance, and suppression of glial cell activation may be one mechanism underlying this phenomenon (Takemoto et al., 2016; Katayama et al., 2018). A study that investigated orexin secretion found that orexin secretion was significantly increased in rats with morphine tolerance; however, YKS administration significantly suppressed it (Katayama et al., 2018). Orexin is a neuropeptide secreted from the hypothalamus. It has an analgesic effect (Yamamoto et al., 2003), but under pathological conditions of chronic pain, the excessive secretion of orexin may disrupt the pain modulatory system. The administration of an orexin receptor antagonist to rats with morphine tolerance, therefore suppressed the decrease in the pain threshold (Erami et al., 2012) and also exerted analgesic effects against acute and chronic pain (McDonald et al., 2016). We also found that YKS suppressed orexin secretion in a dose-dependent manner in healthy rats (Katahira et al., 2017). These findings suggest that the analgesic effect of YKS is partly involved in the inhibition of orexin secretion.

**FIGURE 1:**
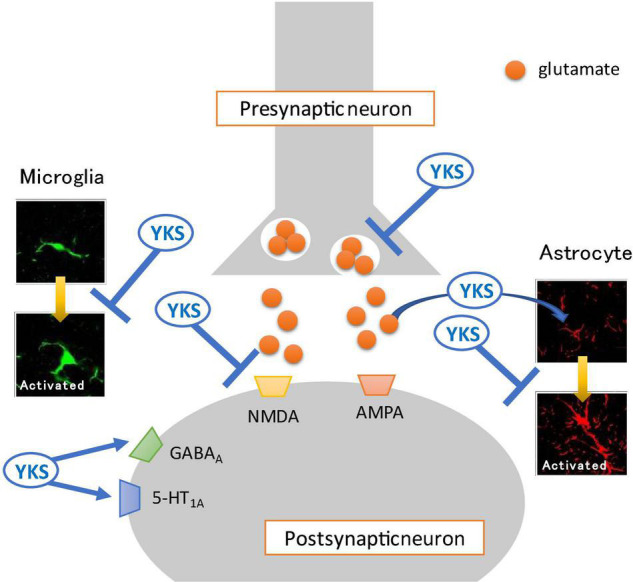
Mechanisms of action of Yokukansan for neuropathic pain. Several different mechanisms of action may act on neurotransmission in the spinal dorsal horn. (1) Attenuation of excessive glutamate release from presynaptic neurons. (2) Promotion of the uptake of glutamate into astrocytes. (3) Antagonistic effect on the glutamate receptor, *N*-methyl-D-aspartate (NMDA) receptor. (4) Agonistic effect on the GABA_A_ receptor. (5) Inhibition of the activation of glia cells (microglia and astrocyte). (6) Agonistic effect on the serotonin 5-HT_1A_ receptor. YKS, Yokukansan; AMPA, α-amino-3-hydroxy-5-methyl-4-isoxazolepropionic acid receptor.

Oxytocin is also a neuropeptide secreted from the hypothalamus and has been reported to have a central-acting analgesic effect (Sun et al., 2018; González-Hernández et al., 2019). YKS administration also increased oxytocin secretion in rats (Kanada et al., 2018). The analgesic effect of YKS may be related to the secretagogue effect of oxytocin. Future studies should be conducted using pain model animals. Figure 1 summarizes the main actions of YKS.

## Conclusion

The multiple ingredients that comprise Kampo formulae exert various beneficial effects. Although the individual pharmacological action of the components might be weak, the combination of these actions confers a holistic effect on intractable pain. This is an important point to consider in future pain treatment strategies. Multiple central sensitizations cause chronic pain; therefore, multi-component drugs, such as Kampo formulae, are more beneficial than seeking a strong analgesic effect with a single agent. In addition, identifying the active ingredients in the drugs used in traditional medicine can lead to the development of new drugs.

## Author Contributions

YT and MS participated in the conception and design. KY and MS wrote the draft. All authors retrieved and reviewed the literature and accepted responsibility for the entire content of this manuscript and approved its submission.

## Conflict of Interest

MS received a research grant from Tsumura & Co. (Tokyo, Japan). The remaining authors declare that the research was conducted in the absence of any commercial or financial relationships that could be construed as a potential conflict of interest.

## Publisher’s Note

All claims expressed in this article are solely those of the authors and do not necessarily represent those of their affiliated organizations, or those of the publisher, the editors and the reviewers. Any product that may be evaluated in this article, or claim that may be made by its manufacturer, is not guaranteed or endorsed by the publisher.

## Abbreviations

SNRI: serotonin noradrenaline reuptake inhibitor
GJG: Goshajinkigan
EJT: Eppikajutsuto
KBG: Keishibukuryogan
GRS: Goreisan
YKS: Yokukansan
JTT: Juzentaihoto
KJT: Keishikajutsubuto
SKT: Saikokeishito
CRPS: complex regional pain syndrome
CCI: chronic constriction injury
PSL: partial sciatic nerve ligation
NMDA: *N*-methyl-D-aspartate.
